# T-cell receptor repertoire of cytomegalovirus-specific cytotoxic T-cells after allogeneic stem cell transplantation

**DOI:** 10.1038/s41598-020-79363-2

**Published:** 2020-12-17

**Authors:** Takashi Toya, Ayumi Taguchi, Kazutaka Kitaura, Fumi Misumi, Yujiro Nakajima, Yuki Otsuka, Ryosuke Konuma, Hiroto Adachi, Atsushi Wada, Yuya Kishida, Tatsuya Konishi, Akihito Nagata, Yuta Yamada, Atsushi Marumo, Yuma Noguchi, Kota Yoshifuji, Junichi Mukae, Kyoko Inamoto, Aiko Igarashi, Yuho Najima, Takeshi Kobayashi, Kazuhiko Kakihana, Kazuteru Ohashi, Ryuji Suzuki, Takeshi Nagamatsu, Noriko Doki

**Affiliations:** 1 Hematology Division, Tokyo Metropolitan Cancer and Infectious Diseases Center, Komagome Hospital, 3-18-22 Honkomagome, Bunkyo-ku, Tokyo, 113-8677 Japan; 2 Department of Obstetrics and Gynecology, The University of Tokyo, 7-3-1 Hongo, Bunkyo-ku, Tokyo, 113-8655 Japan; 3 Department of Gynecology, Tokyo Metropolitan Cancer and Infectious Diseases Center, Komagome Hospital, Tokyo, Japan; 4 Repertoire Genesis Inc., Ibaraki, Japan; 5 Department of Radiation Oncology, Tokyo Metropolitan Komagome Hospital, Tokyo, Japan; 6 Radiation Oncology, Tohoku University Graduate School of Medicine, Sendai, Japan

**Keywords:** Transplant immunology, Viral infection, Immunology, Medical research

## Abstract

Cytomegalovirus (CMV) infection is a major complication during allogeneic stem cell transplantation (allo-SCT). However, mechanisms of adaptive immunity that drive this remain unclear. To define early immunological responses to CMV after transplantation, we using next-generation sequencing to examine the repertoire of T-cell receptors in CD8^+^/CMV pp65 tetramer^+^ cells (CMV-CTLs) in peripheral blood samples obtained from 16 allo-SCT recipients with HLA-A*24:02 at the time of CMV reactivation. In most patients, TCR beta repertoire of CMV-CTLs was highly skewed (median Inverse Simpson’s index: 1.595) and, 15 of 16 patients shared at least one TCR-beta clonotype with ≥ 2 patients. The shared TCRs were dominant in 12 patients and, two clonotypes were shared by about half of the patients. Similarity analysis showed that CDR3 sequences of shared TCRs were more similar than unshared TCRs. TCR beta repertoires of CMV-CTLs in 12 patients were also analyzed after 2–4 weeks to characterize the short-term dynamics of TCR repertoires. In ten patients, we observed persistence of prevailing clones. In the other two patients, TCR repertoires became more diverse, major clones declined, and new private clones subsequently emerged. These results provided the substantive clue to understand the immunological behavior against CMV reactivation after allo-SCT.

## Introduction

Allogeneic hematopoietic stem cell transplantation (allo-SCT) is a curative treatment procedure for many hematological and non-hematological disorders. However, persistent immunodeficiency can evoke various infection complications, including cytomegalovirus (CMV) reactivation and diseases^1^. CMV diseases are a major cause of morbidity and mortality in allo-SCT recipients^2,3^. In addition to CMV diseases, CMV reactivation has also been reported to indirectly increase non-relapse mortality^4,5^. However, the exact mechanisms remain unclear.

Adoptive immunity, especially T-cell immunity, is considered to be important anti-CMV protection. Recently, high-throughput next-generation sequencing (NGS) technology has provided detailed data that comprehensively shed light on the landscape of T-cell receptor (TCR) repertoires^6^. While some studies focused on characterizing the overall picture of anti-CMV TCR repertoires^7–13^ or TCRs after allo-SCT^14–21^, there are few reports using NGS to define the CMV-specific TCR repertoire after allo-SCT^22–24^. Some recent reports have also suggested that shared TCRs are important. They were identified as TCRs that are commonly observed across multiple individuals, and have been reported to exhibit higher functional activity in various populations including transplantation recipients^8,9,25^.

 However, details regarding CMV-specific shared TCRs have yet to be defined. Additionally, it has been reported that the diversity of HIV-specific TCR repertoire and the dominant clones in patients with HIV-1 can change dynamically during the clinical course^26,27^.

 Although it has been proposed that early behavior of CMV-specific immunity after allo-SCT could be important for control of CMV viremia^28^, clonal dynamics of CMV-specific immunity, especially their short-term changes following CMV reactivation, remains to be elucidated.

Human leukocyte antigen (HLA)-specificity adds a further layer of complication to this problem. There have been recent attempts to use T-cell therapy to treat CMV and other viruses^29–35^. However, as TCRs are restricted by HLA-specificity, when planning to perform T-cell therapy with a third-party donor (TPD) rather than a stem cell donor, it is necessary to have a good understanding of the TCR repertoire specific to that HLA type. It is also reported that the persistence of transferred T-cells, which should be important for sustaining anti-CMV immunity, is variable in TPD setting^31^. Therefore, characterization methods for elite TCR clones, which have high reactivity against CMV and are persistent in recipients for a long time, are warranted for more sophisticated adoptive T-cell therapy.

In this study, we used unbiased NGS to characterize the TCR repertoire of CMV-specific cytotoxic T-cells (CMV-CTLs) after CMV infection in 16 patients with HLA-A*24:02, one of the most common HLA types in Japanese and East Asian populations^36^, to define early immunological responses to CMV after transplantation. We found that the CMV-specific TCR repertoire at the time of CMV reactivation was highly skewed and shared TCRs, which had the majority in CMV-CTLs, were commonly detected. We also evaluated the short-term dynamics of TCR repertoires and found that while most patients maintained skewed TCR repertoires, diversity could increase in some patients. These results shed light on the characteristics of prominent TCRs in the specific immune response following allo-SCT, which is suggestive of the potential role of specific clone selection for more sophisticated cellular therapy.

## Results

### T-cell receptor repertoire analysis of CMV-CTLs

**Figure 1:**
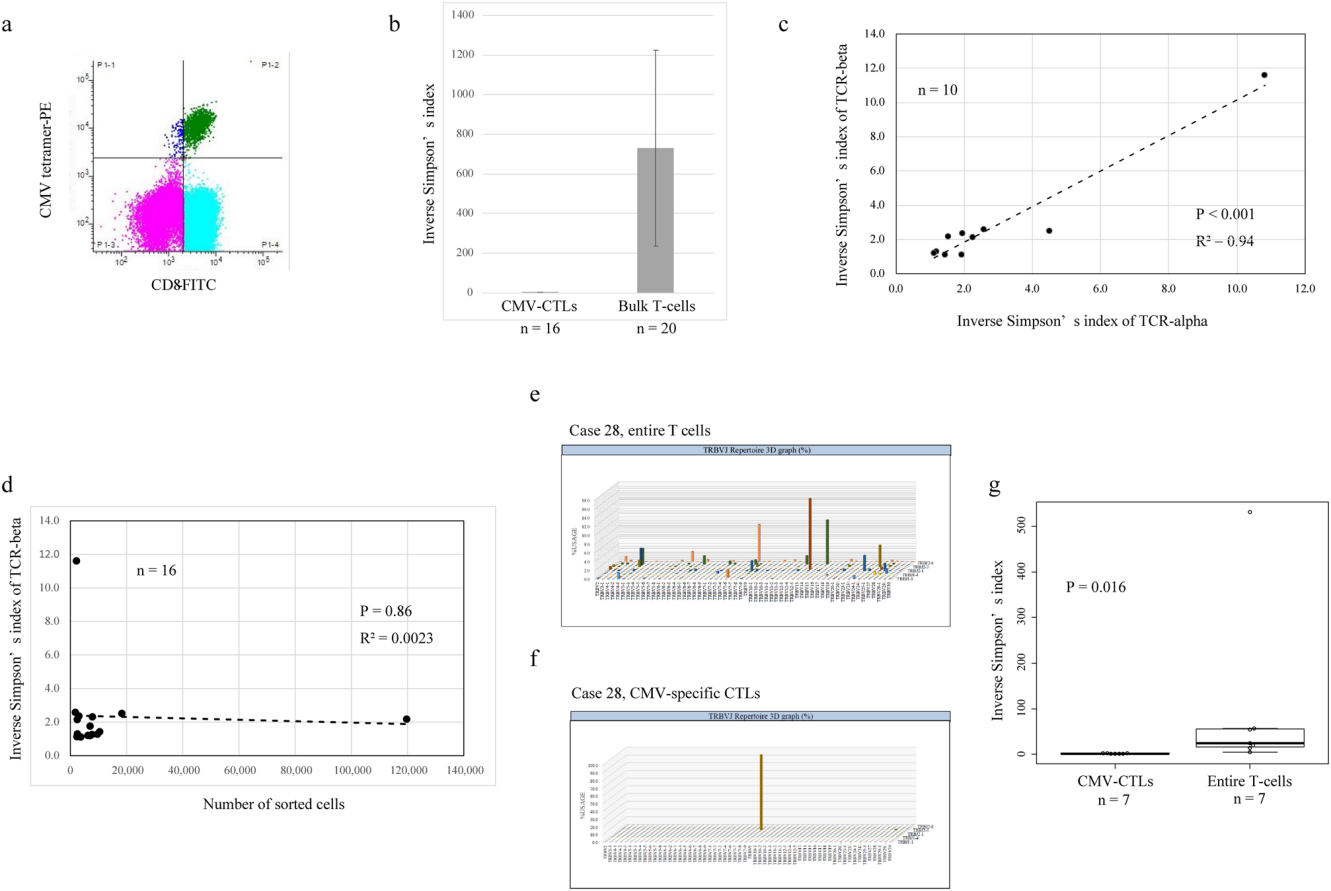
(**a**) Representative image of the gating used for cell sorting. CD8^+^/HLA-A*24-specific cytomegalovirus (CMV) pp65 tetramer^+^ cells were sorted (green fraction, upper-right). (**b**) Box-plot representing the diversity of the T-cell receptor (TCR)-beta repertoire of CMV-specific cytotoxic T-cells (CTLs) (left column) and published data on the diversity of TCR-beta repertoire of entire T-cells (right column)^37^. (**c**) Correlation between the diversity of TCR-alpha and -beta in CMV-CTLs. (**d**) Correlation between the diversity of TCR-beta in CMV-CTLs and the number of sorted cells. (**e**,**f**) A 3D graphical representation of the TCR repertoire in the entire T-cell population and CMV-CTLs. The X and Y axes show TRBV and J segments respectively and the Z axis indicates frequency. (**g**) Box-plot representation of the diversity of the TCR-beta repertoire in CMV-CTLs and the entire T-cell population.

The median number of sorted CD8^+^/CMV pp65 tetramer^+^ cells used for TCR repertoire analysis was 6666 cells/sample (range 1826–119,706 cells/sample, Fig. 1a) and the median number of assigned reads was 127,998 reads/sample (range 67,181–460,219, Supplementary Table S1). The diversity of TCR repertoire of CMV-CTLs at the time of CMV reactivation was quite low in most patients, and this was represented by a low median Inverse Simpson’s index score of 1.595 (95% confidence interval 0.990–3.688) when compared to scores of previously published TCR diversity of bulk T-cells in peripheral blood (PB) of healthy individuals (729.7 ± 493.9, Fig. 1b)^37^.

Both TCR-alpha and TCR-beta were analyzed in 10 patients and their diversity showed good correlation (P < 0.001, Fig. 1c). No obvious association between the number of sorted cells and TCR diversity was observed (P = 0.86, Fig. 1d). The TCR-beta repertoire of the entire T-cell population was analyzed in seven patients (median Inverse Simpson’s index: 24.7) and this diversity was significantly higher (P < 0.001) than that of CMV-CTLs in each patient (median Inverse Simpson’s index: 1.43, Fig. 1e–g). After allo-SCT, the TCR-beta repertoire diversity of the whole T-cell population was lower than what has previously been reported for normal populations^38^.

As stated above, we analyzed the TCR repertoire of CMV-CTLs as well as of the entire T-cell population at CMV reactivation in 7 patients. In 3 patients among them, the TCR repertoire of CMV-CTLs and the entire T-cell population 2–4 weeks after CMV reactivation was also evaluated. In these 10 samples, we identified 30 clones which comprised more than 1% of the CMV-CTLs in each sample and, among them, 24 clones were also identified in the entire TCR repertoire in each sample. When we calculated the frequency of each CMV-specific clone in the entire T-cell population by multiplying the frequency of CMV-CTLs in entire T-cells and frequency of each clonotype in CMV-CTLs, calculated frequencies were generally comparable to the actual frequency of CMV-CTLs. Nevertheless, in some cases, the calculated frequency was much lower than the actual frequency (Supplementary Table S2).

**Figure 2:**
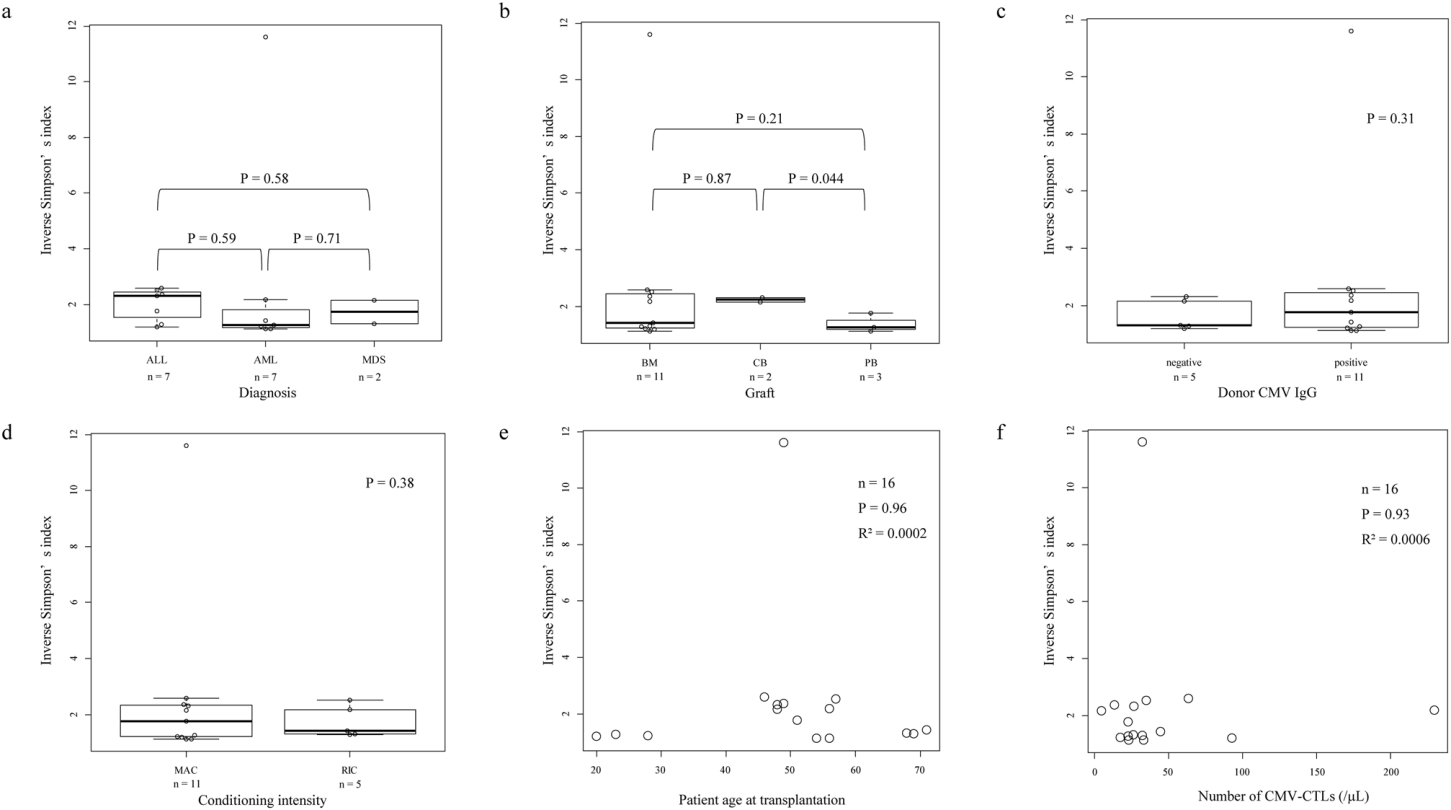
Associations between the diversity of T-cell receptor (TCR)-beta repertoire of cytomegalovirus-specific cytotoxic T-cells (CMV-CTLs) and clinical characteristics. Association between TCR repertoire diversity and (**a**) diagnosis of hematological diseases, (**b**) graft source, (**c**) donor CMV serology, (**d**) conditioning intensity, (**e**) recipient age at transplantation, and (**f**) the number of CMV-CTLs at CMV reactivation.

We performed analyses to evaluate possible relationships, but found no significant associations between clinical parameters with the TCR repertoire diversity of CMV-CTLs (Fig. 2, Supplementary Table S1). There were no apparent differences in diversity and dominant clones between CMV-IgG positive and negative donors.

### Characteristics of CMV-CTL TCRs

**Figure 3:**
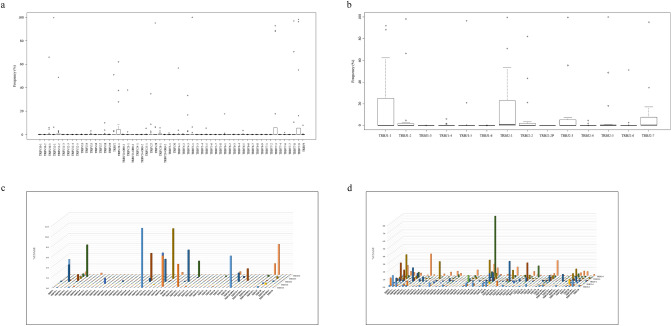
Characteristics of cytomegalovirus (CMV)-specific T-cell receptors (TCRs). (**a**,**b**) Boxplot representing TRBV (**a**) and TRBJ (**b**) usage of CMV-CTLs. (**c**,**d**) 3D graphical representation of the TCR repertoire of CMV-CTLs (**c**) and entire T cells (**d**) at the time of CMV reactivation. The X and Y axes indicate TRBV and TRBJ segments, respectively, and the Z axis indicates frequency.

Next, we analyzed TRBV/TRBJ family characteristics in CMV-CTLs (Fig. 3a,b). In accordance with the low diversity of CMV-specific TCRs, the median frequency of most V and J families was close to 0% while some dominant clones were detected in a few cases. The frequencies of some clonotypes with specific TRBV and TRBJ families (TRBV4-2, TRBV5-4, TRBV5-6, TRBV6-4, TRBV6-5, TRBV6-6, TRBV7-2, TRBV7-6, TRBV9, TRBV10-1, TRBV10-2, TRBV12-4, TRBV19, TRBV30 and TRBJ1-3) were significantly lower in CMV-CTLs compared to entire T-cells, while frequencies for TRBV and TRBJ families were not observed in CMV-CTLs.

We also evaluated the combination of V and J family usages in CMV-CTLs (Fig. 3c) and entire T-cells (Fig. 3d). Although there seemed to be some discrepancy in V-J usage between CMV-CTLs and entire T-cells, no statistically significant difference was observed in the frequency of each V–J combination between CMV-CTLs and entire T-cells.

### Shared TCRs and similarity analysis

**Figure 4:**
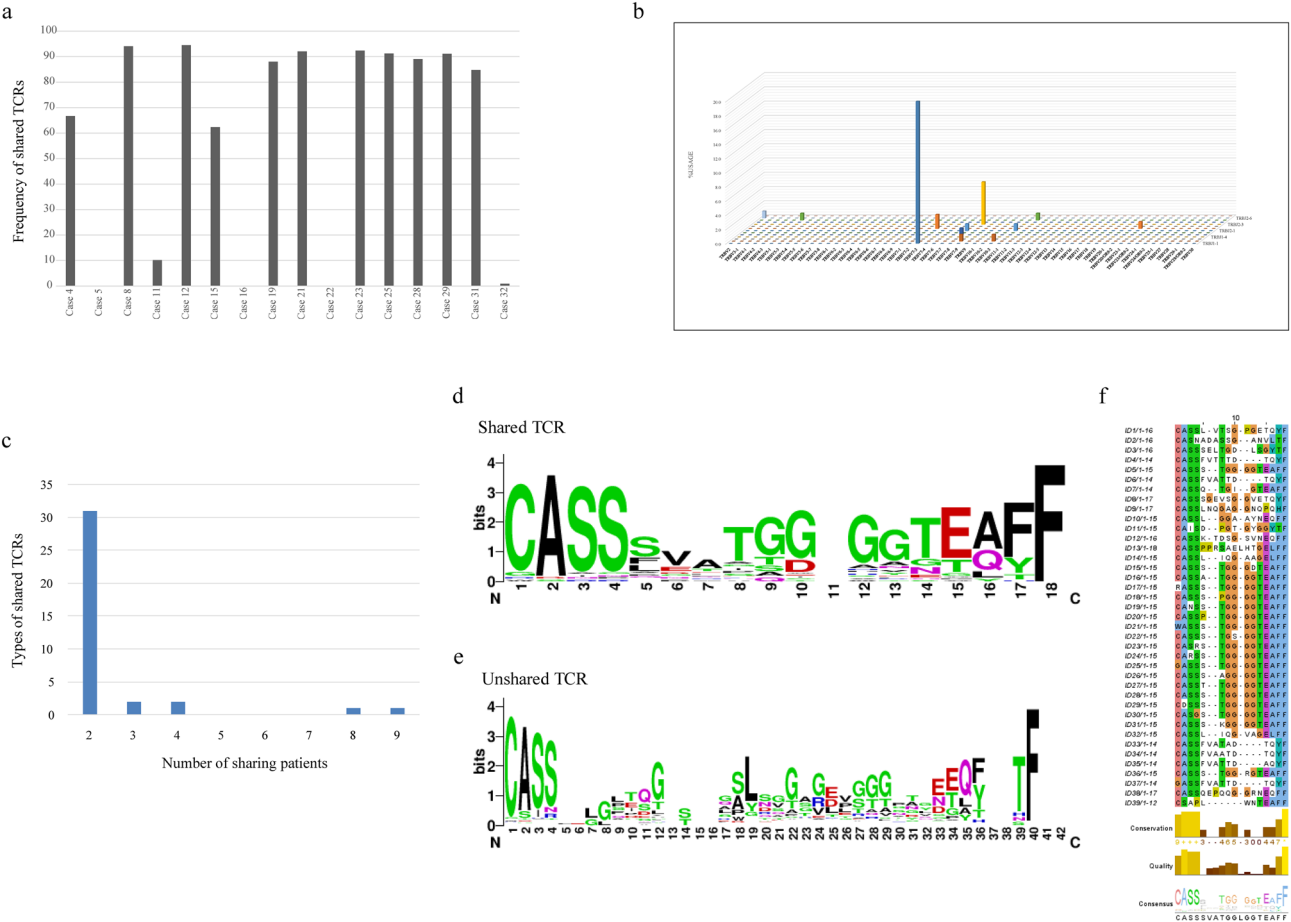
Characteristics of shared T-cell receptors (TCRs) of cytomegalovirus-specific cytotoxic T-cells. (**a**) Frequency of shared TCRs among CMV-CTLs in each patient. (**b**) TRBV and TRBJ usage of shared TCRs. The X and Y axes indicate TRBV and TRBJ segments, respectively, and the Z axis indicates the number of shared TCR types. (**c**) The number of TCRs and number of different patients that share each sequence. (**d**,**e**) Representation of multiple sequence alignments for complementarity determining region 3 (CDR3) in (**d**) shared and (**e**) unique TCRs. The relative size of the amino acid letters in the logo diagram represents the frequency of representation for each amino acid. (**f**) List of amino acid sequences in CDR3 for shared TCRs.

We next focused on the frequency, V and J usage, and complementarity determining region 3 (CDR3) sequences of shared TCR-beta in CMV-CTLs, and 37 types of shared TCRs were identified (1.2% of the 2992 type unique reads). Interestingly, 15 of the 16 patients had at least one shared TCR (Supplementary Table S3), and shared clonotypes were dominant in 12 patients (Fig. 4a). With regard to TRBV and TRBJ usage, there was a substantial bias of usage, with 20 of the 37 shared TCR being TRBV7-3/TRBJ1-1, and 6 of them being TRBV7-9/TRBJ2-3 (Fig. 4b). However, most of these TCRs were shared by only a few patients. Of note, we also identified two clonotypes, namely TRBV2/TRBJ2-6/CASNADASSGANVLTF and TRBV11-2/TRBJ2-5/CASSLVTSGPGETQYF, which were shared by about half of the 16 patients (detected in 8 and 9 patients respectively, Fig. 4c; Supplementary Table S1).

Similarity analysis using MUSCLE algorithm and Jalview software showed that CDR3 sequences of shared TCRs had a higher degree of similarity compared to those of non-shared TCRs (Fig. 4d–f, Supplementary Tables S3–S5).

### Clonal dynamics of TCR repertoire

**Figure 5:**
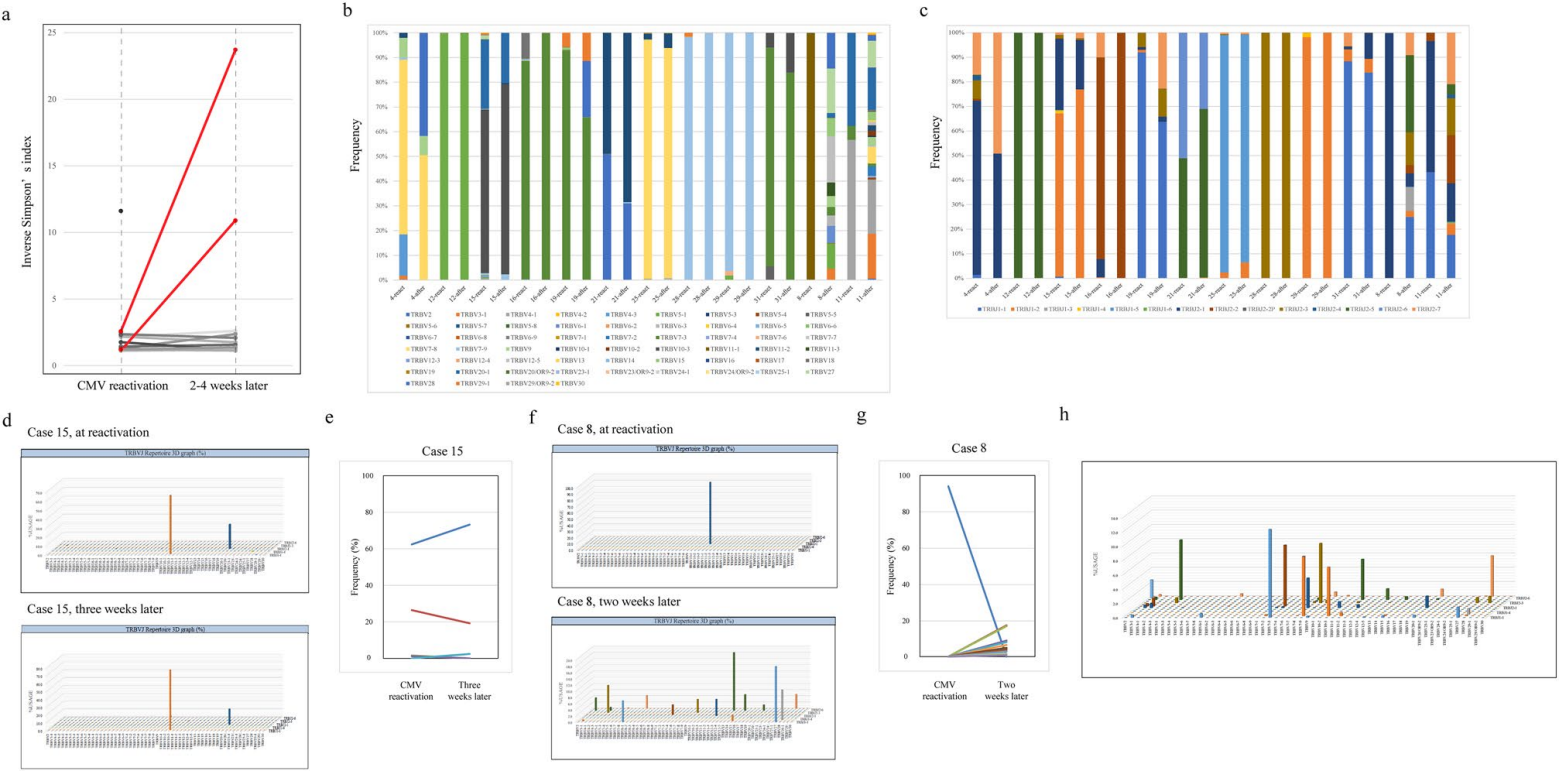
Clonal dynamics of CMV-CTLs. (**a**) T-cell receptor-beta repertoire of cytomegalovirus-specific cytotoxic T-cells (CMV-CTLs) and its changes over time. Two cases with remarkable changes in diversity are highlighted in red. (**b**,**c**) Frequency of each TRBV (**b**) and TRBJ (**c**). (**d**) A 3D graphical plot of T-cell repertoire of CMV-CTLs at the time of CMV reactivation (above) and 3 weeks after (below) for Case 15. (**e**) Frequency and time course for each CMV-CTL clonotype in Case 15. Clonotypes that accounted for ≥ 1% were represented. (**f**) 3D graphical plot of T-cell repertoire of CMV-CTLs at the time of CMV reactivation (above) and 2 weeks after (below) for Case 8. (**g**) Frequency and time course for each CMV-CTL clonotype in Case 8. Clonotypes that accounted for ≥ 1% were represented. (**h**) A 3D graphical representation of the TCR repertoire of CMV-CTLs 2–4 weeks after CMV reactivation.

When we sequentially analyzed TCR beta repertoires in 12 patients to evaluate clonal transition, shared clonotypes were consistently detected in 10 of 11 patients with shared TCR. Additionally, two patterns of subsequent clonal behavior were detected in the TCR repertoire of CMV-CTLs. In 10 patients, variants of clones persisted and TCR repertoires of CMV-CTLs remained oligoclonal (Fig. 5a–c, Supplementary Fig. S1). Frequency of V-J pairs at each time point and transition of CMV-CTL clonotypes in case 15 are shown as an example of such cases (Fig. 5d,e). However, in the two other patients, TCR repertoires of CMV-CTLs became more diverse. The Inverse Simpson’s index increased from the time of reactivation to 2–4 weeks after (1.129–10.90 in one patient and 2.583–23.69 in the other), major clones markedly decreased, and new private clones subsequently appeared (Fig. 5a–c, Supplementary Figure S1). V–J usage at each time point and transition of CMV-CTL clones in case 8 are shown as an example (Fig. 5f,g). A polyclonal pattern, or a diverse TCR repertoire in CMV-CTLs at the time of reactivation [n = 1] or after 2–4 weeks [n = 2] was detected in 3 of 16 patients. This pattern was observed exclusively in patients who were administered corticosteroid (prednisolone, 20–30 mg/day) upon CMV reactivation (42.9% vs. 0.0%, P = 0.063). The 3D figure of the TRBV and TRBJ combination also indicated that some CMV-specific TCRs identified at CMV reactivation disappeared after 2–4 weeks, and markedly few novel TCRs appeared with most of the major clones detected 2–4 weeks after CMV reactivation already present at CMV reactivation (Figs. 3c, 5h).

## Discussion

In this study, we used unbiased NGS to characterize the TCR repertoire of CMV-CTLs in 16 patients of type HLA-A*24:02 who underwent allo-SCT and suffered from CMV infection. Just as Miyama et al. demonstrated that CMV-CTLs in healthy individuals were oligoclonal even after in vitro stimulation^8^, this study also showed similar results in vivo in 12 patients who had undergone allo-SCT. These results are also consistent with a previous report by Link et al. that evaluated the TCR repertoire of CMV-CTLs in four patients of type HLA-A*02:01 after allo-SCT^23^. In this study, a larger number of patients was analyzed and results indicated that shared TCRs were common within allo-SCT recipients with CMV reactivation. These data were consistent with data from the previous study which reported that repertoire overlap was significantly driven by CMV but otherwise remained markedly limited^39^. Although frequency of shared TCRs and the number that is shared can depend on cohort size and sampling depth^40^, the higher frequency of shared clonotypes and markedly similar CDR3 sequences observed suggested that they may play important roles. In this study, we constructed the first TCR repertoire database from CMV-CTLs that are specific for HLA-A*24:02. In VDJdb^41^, only 38 sequences of 4 patients from 2 studies^22,42^ were registered as TCRs of HLA-A*24:02-specific anti-CMV pp65 tetramer-positive CTLs. Although our shared TCR sequences were not included in this database, it might be due to a lack of cases, and more data should be accumulated to clarify the characteristics and distribution of these sequences. Our results showed that several specific TCRs were consistently and exclusively detected after CMV reactivation, suggesting that these TCRs had a crucial role in controlling CMV infection.

We compared the TCR repertoire of CMV-CTLs and entire T-cells, thereby demonstrating that the frequency of CMV-specific TCRs in entire T-cells were comparable to the frequency which was calculated from frequency of CMV-CTLs in entire T-cells and frequency of specific TCRs in CMV-CTLs in many cases. Additionally, in sequentially analyzed cases, various clones were consistently identified. While these results suggested the robustness of such TCRs and our analytical methods, there were discrepancies. The exact reason was unclear, but perhaps such clones were not activated well, and most of the clones with deviation had exceedingly low frequency. Therefore, coverage might be insufficient to detect such minor populations. The affinity of each TCR and CMV pp65 tetramer could also affect the results, as it was previously reported that affinity could influence the sorting efficiency^9,24^.

In this study, we observed an interesting change in TCR repertoire diversity and clonal shift of CMV-CTLs in some patients. However, the exact mechanisms and clinical significance of this phenomenon are unclear. Although the TCR repertoire generally remains stable^43,44^, a change of TCR repertoire diversity and clonal dynamics of antigen-specific T-cells have also been reported in some contexts, including allo-SCT settings^22,24^. For example, Poiret et al. analyzed the behavior of CMV-CTLs after allo-SCT and showed that the population of CMV-CTLs could change over time^24^; similar results were also reported by Nakasone et al.^22^. Ramien et al. analyzed the TCR repertoire of patients with multiple sclerosis and reported that TCR clones became significantly more diverse with the amelioration of disease symptoms during pregnancy, with possible induction of immunotolerance in the expectant mothers^45^. Costa et al. reported that in HIV-1 patients, the diversity of the HIV-specific TCR repertoire could change and that changes in diversity of the antigen-specific repertoire and magnitude of CD8^+^ T-cell responses were inversely correlated^26^. Other reports have also suggested that dominant and prominent TCRs, rather than a diverse TCR repertoire, appeared to be clinically important in specific immunity^22,46^. Persistence of dominant TCRs might also be important as we observed that increases in TCR diversity were accompanied by decreases in dominant clones. Considering that all three patients in our study who had diverse TCR repertoires were systemically administered corticosteroid therapy, it is likely that severe immunosuppression may have hampered the stable proliferation of CMV-CTLs and may have thus facilitated clonal shift. Additionally, while previous studies have often focused on clonal dynamics over a longer period, this study showed that the composition of specific T-cell clones could undergo change in a short period of time^22^.

Some reports indicated clinical efficacy of cellular therapy for CMV diseases after allo-SCT^30–32^. However, although infused T-cells derived from stem cell donors were generally persistent at least for some years, in many cases, T-cells from TPD disappeared soon after infusion probably because they were rejected by the allograft derived from the stem cell donor^31,47^. The longevity of infused T-cells should be important for the control of CMV because late and/or recurrent CMV infection is common among allo-SCT recipients^48^. Considering that late CMV infection often occurs after allo-SCT^49,50^, administration of persistent antiviral T-cells may be superior to antiviral drugs as patients may be spared the side effects and costs. The exact reason underlying TCR longevity remains unclear, but it is reported that antigen exposure has a crucial role for T-cell expansion and persistence^51^; therefore the reactivity of each TCR against the relevant epitope may be important, although other strategies such as repeated infusion, use of allo-SCT donor T-cells, post-transfer vaccination^52^, and application of chimeric antigen receptor-modified T-cells^53^ may also lead to longevity of infused T-cells. Our results suggest that characterization of “elite” TCR clones is warranted for more sophisticated adoptive T-cell therapy. It is presumed that public TCRs detected in many individuals may have higher compatibility of variable patients, may exhibit lower immunogenicity, and can be a good candidate for adoptive T-cell therapy from TPD. We believe that these results provide a substantive clue to understand the immunological behavior against CMV reactivation after allo-SCT and that the selection of appropriate TCRs could be important for the development of more sophisticated cellular therapy against CMV.

Our study has several limitations. Firstly, the functions of the TCRs were not evaluated in this study. As TCR repertoire analyses are purely quantitative analyses, identification of TCR alpha/beta pairs and a comprehensive qualitative evaluation is necessary for functional assessment. In light of previous reports that have indicated higher avidity and activity of shared TCRs in several contexts^8,9,25^, the shared clonotypes that were identified could have prominent immunological functions. Additionally, as indicated by Poiret et al. through the analysis of three different HLA-A*02:01-specific CMV tetramers, the affinity of CMV-CTLs may be a promising biomarker for evaluation of the function of identified TCRs^24^. Secondly, we could not evaluate whether CMV-specific TCR repertoire behavior after HLA-mismatched transplantation was different from HLA-matched transplantation because all patients in our study underwent HLA-A matched allo-SCT, as with previous studies^8,9,11,12^. Considering recent increase of haploidentical transplantation^54^, characterization of immune reconstitution after HLA-mismatched transplantation is warranted. Thirdly, as we only analyzed patients who suffered from CMV reactivation, the difference of TCR repertoire between those and patients who did not experience CMV reactivation was unclear. Nakasone et al. reported that an oligoclonal CMV-specific TCR repertoire was not a hallmark of reactivation^55^, and it seemed less important to identify clinically functional TCRs against CMV. We speculate that the absence of relevant TCRs in patients who suffer from repeated and protracted CMV infection/diseases, rather than patients who do not experience CMV reactivation, may be better circumstantial evidence for the clinical importance of such clones. However, unfortunately most of such patients were administered a high dose of corticosteroid and marked lymphopenia prevented us from reliable analyses because not enough RNA could be obtained for repertoire analysis after tetramer sorting. Perhaps this technical barrier may be overcome through single-cell or ultra-low-input sequencing. Finally, although CMV reactivation was reported to be associated with lower relapse risk especially in patients with AML^3,56,57^, and Yew et al. suggested the association between TCR repertoire after allo-SCT and relapse/graft-versus-host disease (GVHD)^18^, as only one patient with ALL suffered from relapse in this patient cohort, it was not possible to identify any associations between CMV reactivation, TCR repertoire, and relapse. Additionally, GVHD was not apparently associated with CMV-specific TCR repertoire diversity or the existence of specific clones in this study. This was probably due to the small sample size or timing of sample collection considering the fact that Buhler et al. reported that clonality one year after allo-SCT was not associated with risk of clinical events^39^. Future studies with more cases and/or sequential evaluation are therefore warranted.

In conclusion, the TCR repertoire of CMV-CTLs after allo-SCT is oligoclonal in most cases at the time of CMV reactivation, but may undergo short-term dynamic changes in a minority of patients. Shared TCRs were frequently detected in allo-SCT recipients and exhibited high sequence similarity. Functional tests are warranted to identify key TCRs that may guard against CMV.

## Methods

### Patients and transplant procedures

Sixteen patients with type HLA-A*24:02 who underwent allo-SCT at our hospital and experienced CMV reactivation were included in this study. One patient underwent a second allo-SCT. For all other patients, this was their first allo-SCT. Intensity of each conditioning regimen was classified, as previously defined^58^. Donors that had 8/8 allele matches for HLA A, B, Cw and DRB1 were considered to be HLA-matched. Calcineurin inhibitor and methotrexate (short-term) were used for prophylaxis of GVHD. Surveillance of CMV pp65 antigenemia was started from time of neutrophil engraftment, and was monitored weekly until ≥ 100 days after allo-SCT. CMV reactivation was defined as being positive for CMV antigenemia by C7-HRP testing. No patient received prophylactic therapy for CMV. Peripheral blood samples were collected either weekly or fortnightly from time of neutrophil engraftment until approximately 100 days after allo-SCT, and the samples collected immediately after CMV reactivation and 2–4 weeks later were subsequently analyzed.

Written informed consent was obtained from every patient. This study was performed in accordance with the Declaration of Helsinki and was approved by the Institutional Review Board of Tokyo Metropolitan Cancer and Infectious Diseases Center, Komagome Hospital, Tokyo, Japan (number 1966).

### Patient characteristics

Sixteen transplant recipients were included in this study conducted from March to October 2018. Their clinical characteristics are summarized in Table 1 and Supplementary Table S1. The median age of the recipients was 50 years (range 20–71). Patients were being treated for acute myeloid leukemia (n = 7), acute lymphoblastic leukemia (n = 7), or myelodysplastic syndromes (n = 2). Eleven patients received bone marrow, three patients received PB stem cells, and two patients received unrelated cord blood transplantation. Two donors were related to recipients and 14 were unrelated. Eleven patients underwent a myeloablative conditioning regimen and five underwent reduced intensity conditioning. Of the 16 patients, 9 received grafts from a HLA-matched donor. Only one patient who received a bone marrow transplant from an unrelated, HLA-DRB1 mismatched donor, was prophylatically administered anti-thymocyte globulin (Thymoglobulin, 2.5 mg/kg) for GVHD. All recipients were CMV IgG-positive and 11 donors were CMV-seropositive. There was no CMV reactivation/disease observed in any patient prior to allo-SCT. The first clinically significant CMV infection was observed within a median of 35 days (range 16–55) post-transplantation. The median percentage of sorted CD8^+^/CMV pp65 tetramer^+^ cells upon CMV reactivation was 0.63% (range 0.05–6.56). All patients were preemptively treated with ganciclovir, valganciclovir, or foscarnet, and no patient developed CMV disease. Nine patients suffered from acute GVHD (grade 1 in seven and grade 2 in two patients) and eleven developed chronic GVHD (mild in seven and moderate in four patients) after allo-SCT.

**Table 1:** Patient characteristics.

Median age at transplant, in years (range)	50 (20–71)
**Sex**	
Male	6
Female	10
**Diagnosis**	
Acute myeloid leukemia	7
Acute lymphoblastic leukemia	7
Myelodysplastic syndromes	2
**Donor, donation**	
Related, bone marrow	1
Unrelated, bone marrow	10
Related, peripheral blood	1
Unrelated, peripheral blood	2
Unrelated, cord blood	2
**Human leukocyte antigen**	
Matched donor	9
Mismatched donor	7
**Conditioning intensity**	
Myeloablative	11
Reduced intensity	5
**Recipient CMV IgG**	
Positive	16
Negative	0
**Donor CMV IgG**	
Positive	11
Negative	5
**GVHD prophylaxis**	
Tacrolimus and short-term methotrexate	14
Cyclosporine and short-term methotrexate	2
**T-cell depletion**	
Anti-thymocyte globulin	1
None	15
**Median duration from SCT to first CMV reactivation in days (range)**	39 (16–55)
**Median CMV-CTL number at CMV reactivation, per µL (range)**	29.47 (4.65–229.6)

*CMV* cytomegalovirus, *CTL* cytotoxic T-cell, *SCT* stem cell transplantation.

### Flow cytometry analysis and cell sorting

Mononuclear cells were purified from whole PB (7 mL) by density gradient sedimentation using Ficoll-Paque PLUS (GE Healthcare Life Science, Marlborough, MA). Tetramer and anti-CD8 antibody staining was performed as previously described^8^. Briefly, peripheral blood mononuclear cells (PBMC) were stained with fluorescein isothiocyanate (FITC)-conjugated anti-CD8 (BD Bioscience, San Jose, CA) and phycoerythrin (PE)-conjugated HLA-A*24:02-specific anti-CMV pp65 tetramer (MBL, Nagoya, Japan) in accordance with manufacturer’s instructions. FITC^+^/PE^+^ PBMCs were considered CMV-CTLs, and were sorted using the FACSMelody (BD Biosciences, San Jose, CA, USA) cell sorter (Fig. 1a). Samples that were not stained for tetramer were used as negative controls for non-specific tetramer staining. We mention that 7-AAD or other staining solution to remove dead/damaged cells was not used because marked lymphocytopenia and a low FITC^+^/PE^+^ fraction incentivized us to use a minimal amount of antibodies. Our preliminary experiments suggested that after gating lymphocytes and eliminating doublet cells using FSC-A/FSC-H and SSC-A/SSC-H, CD8^+^/7-AAD^+^ cells were infrequent (data not shown).

### TCR repertoire analysis

Semi-quantitative analysis of the TCR repertoire was performed using high-throughput NGS, as previously described^8^. Briefly, total RNA was extracted from PBMC or sorted FITC^+^/PE^+^ cells using the RNeasy Plus Universal Mini Kit (Qiagen, Hilden, Germany) in accordance with manufacturer’s instructions. Quality of RNA was verified using the Agilent 2200 TapeStation (Agilent Technologies, Palo Alto, CA) and 4 ng of RNA was converted to cDNA using the BSL-18E primer containing oligo d(T)18 and a NotI restriction site. Double-stranded cDNA was synthesized with SuperScript III Reverse Transcriptase (Thermo Fisher Scientific, Waltham, MA, USA) and blunt ends were created with T4 DNA polymerase (Invitrogen). P10EA/P20EA adaptors were ligated to the 5′ end of the cDNA, and cDNA was digested with NotI. After removal of adaptors and primers, a second PCR was performed using TRA- or TRB-constant region-specific and P20EA primers. Following the second PCR, a third PCR was performed using the same conditions with Illumina adaptors for constant region-specific and P20EA primers, and the products were analyzed by high-throughput sequencing using an MiSEQ platform (Illumina, San Diego, CA, USA). Sequences with low quality scores were discarded and only TCR clonotypes whose reads were more than 9 in each patient were analyzed. TCR repertoire was analyzed using bioinformatics software from Repertoire Genesis Incorporation (Ibaraki, Japan). As previously described, TCR clonotypes with the same TRBV/TRBJ gene segments and CDR3 amino acid sequences that were observed in ≥ 2 patients were considered to be shared^8^. Diversity of TCR repertoire was estimated using the Inverse Simpson’s index (1/λ), which was calculated with the formula below:

$$1/\uplambda =\frac{1}{{\sum }_{i=1}^{S}\left(\frac{{n}_{i}\left({n}_{i}-1\right)}{N(N-1)}\right)}$$

where N represents the total number of sequence reads, n_*i*_ is the number of the *i*th unique sequence read, and *S* is the species number of unique sequence reads^37^. A greater score indicates higher diversity. We randomly selected 30,000 reads per sample to standardize the number of TCRs in every sample, and random sampling was repeated 100 times^59^. Median values of the index were used for comparing the diversity of TCR repertoires. A TCR-beta repertoire was considered as polyclonal when 1/λ was higher than the 95% confidence interval of 1/λ of CMV-CTL TCR-beta at CMV reactivation. Multiple alignments of CDR3 sequences were performed using MUSCLE (multiple sequence comparison by log-expectation)^60^, and mutual similarity was analyzed with Jalview software^61^. Illustrations of consensus sequences were created with WebLogo^62^.

### Statistical methods

Differences in numerical and categorical variables were compared using a t test and Fisher’s exact test, respectively. The level of statistical significance was set at *P* value < 0.05. Statistical analyses were performed using R (version 3.5.0; R Foundation for Statistical Computing, Vienna, Austria).

## Supplementary Information

Supplementary Figure S1.

Supplementary Tables.

## Data Availability

*Accession codes* Sequence data for TCR repertoire analyses have been deposited in the GenBank/EMBL/DDBJ sequence read archive (SRA) under the accession code DRA010029.
