# Supplementary figures and images for: T-cell receptor repertoire of cytomegalovirus-specific cytotoxic T-cells after allogeneic stem cell transplantation
Source: Sci Rep. 2020 Dec 17;10:22218. doi: 10.1038/s41598-020-79363-2 (PMC7747720; doi:10.1038/s41598-020-79363-2)

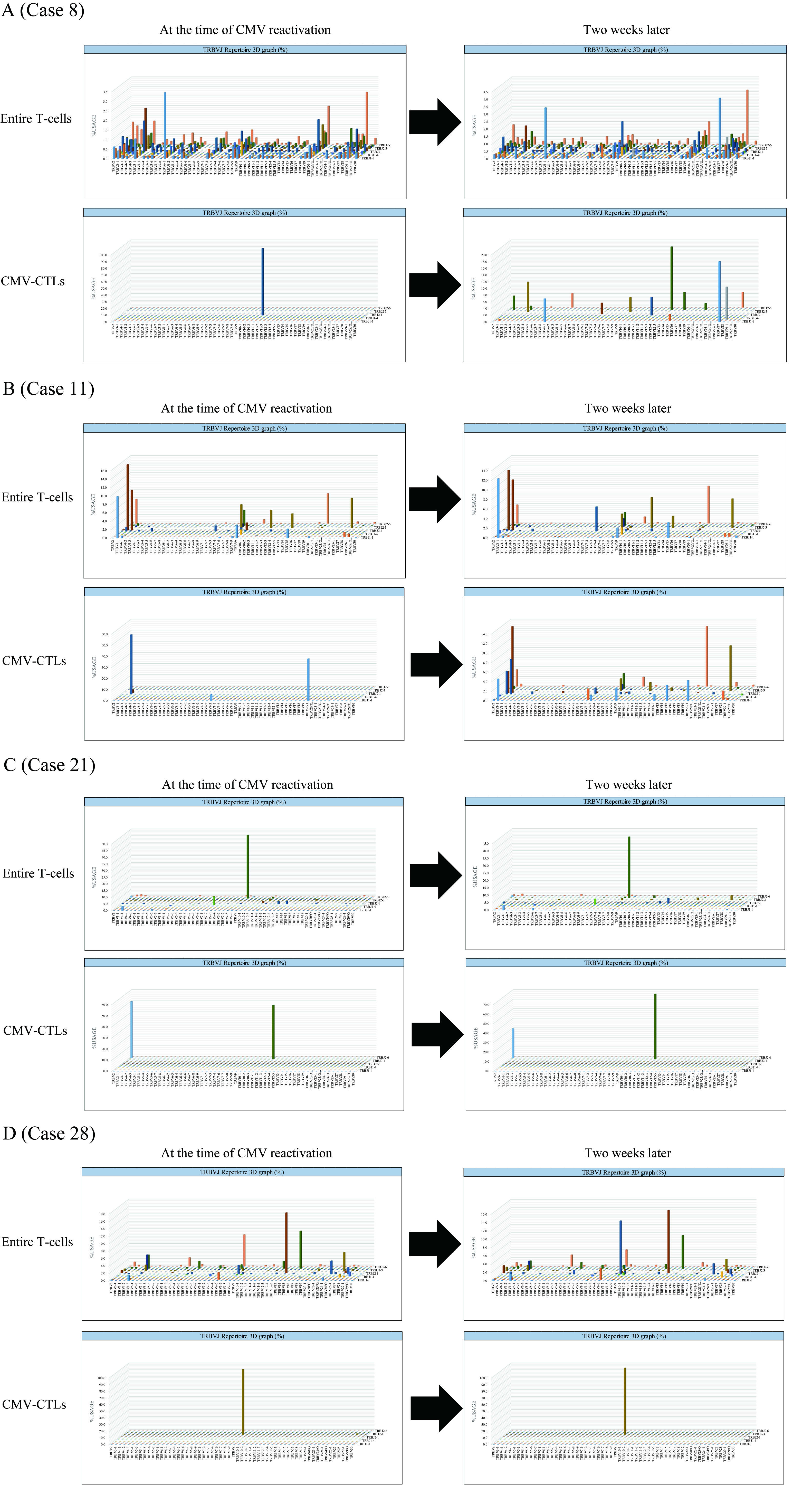

Supplement: Supplementary file 1 — Supplementary Figure S1. [file 41598_2020_79363_MOESM1_ESM.tif]
